# Supplementary material for: Regulatory regions in natural transposable element insertions drive interindividual differences in response to immune challenges in Drosophila
Source: Genome Biol. 2021 Sep 14;22:265. doi: 10.1186/s13059-021-02471-3 (PMC8439047; doi:10.1186/s13059-021-02471-3)
Supplement: Supplementary file 9 — Additional file 9. Review history. [file 13059_2021_2471_MOESM9_ESM.docx]

**Review history**

**First round of review**

**Reviewer 1**

The article entitled "Regulatory regions in natural transposable element insertions drive interindividual differences in response to immune challenges in Drosophila" by Ullastres et al. reports the identification of polymorphic transposable element (TE) insertions that induce changes in immune gene transcript levels and allow an increase in the tolerance of Drosophila melanogaster to infection by Pseudomonas bacteria. This study is very interesting in the context that the determinism of natural variability in pathogen response is largely not understood.
Demonstrating a physiological role to a TE is usually not an easy task because of the difficulty to disentangle the role of the genetic background. In this article, using a variety of sophisticated molecular approaches (in vivo enhancer assays and CRISPR/Cas9 mediated deletions), the authors provide a clear demonstration that some TE insertions may participate in the variability observed regarding host response to pathogens. In addition, their results are very interesting because they illustrate the wideness of the spectrum of TE impacts on host genomes: from none to silencers or enhancers.

The aim and rationale are very clear, the methods are usefully very detailed, and all data are provided as Additional files. The methods are appropriate and all necessary controls are included. The conclusions are adequately supported by the data shown. This study is of interest for a broad audience in the genomics field.

I have a few questions and comments listed below.

   1. I understand that TE insertions in regions with recombination rate = 0 were discarded (line 493); however, how were "regions with high recombination rate" (line 110) defined ? What threshold was used ?
     
   2. Additional file 1B: I don't understand the discrepancy between "number of strains with the TE" and "number of PCR positive strains".
     
   3. Additional file 1D, legend: "Functional (instead of funcional) information of nearby (instead of neaby) genes"
     
   4. Table 1: it would be nice also to tell whether TE insertions are full-length, and when they aren't, to which part of the sequence they correspond.
     
   5. Fig1 (and corresponding text): I find it a little misleading that upregulated and downregulated mutants are treated together. I suggest using separate panels or clearly labeling them.
     
   6. Additional file 3: I could not find the correspondence for the different backgrounds. I guess the solution is in Additional file 6A, but it should be clarified.
     
   7. Figure 3B: labels are missing for the x axis.
     
   8. Line 202 "Candidate adaptive TEs associated… histone mark H3K9me3" section.
      The outputs of statistical tests are missing for the ChIP experiments.
      From Figure 2, FBti0020057 is associated both with increased gene expression (CG15829) and decreased gene expression (CG8628); this should be clarified in this section. In addition, from fig 2, it appears that it is FBti0018868 that is associated with TM4SF; therefore, I don't understand why it is FBti0020057 that is tested with the promoter region of TM4SF in the reporter gene assay.
     
   9. Line 221: why "two of the four"? From Fig2, five and three TEs are associated with high allele-specific expression in control and infection conditions, respectively.
     
   10. Line 228: "promoter regions" : were these regions functionally validated as promoters or are they merely upstream, close to the genes? It is not an issue, but it should be clarified.
     
   11. Figure 4B: half of the graph seems to be missing.
     
   12. Figure 5C: Based on the text line 282, "flies with FBti0019386 overexpressed Bin1 compared to flies within this insertion (p-value < 0.001)". However, significance stars on the figure seem to correspond to the comparison Bin1 total vs red transcript.

   13. ASE experiments: although the use of two different genetic backgrounds strengthens their conclusions, the authors should clearly mention that the insertion of the TE is not the only difference between the two alleles of the heterozygote, and therefore they cannot completely exclude that the TE insertion is not causative for the differences in expression levels. They explain that they have taken this issue into account in the M&M section (line 611), but this point deserves to be discussed more explicitly in the main article.

   14. ASE experiments: I acknowledge that the number of genes is too small for statistical analyses, however, are there trends for associations of the direction of the change in gene expression with the position of the TE relative to the gene (eg upstream vs downstream, near vs far away, etc.) ? Or with the class of the TE ?
     
   15. Line 336: "While these studies found… we found a significant higher proportion". Such a result may also be due to these genes being immune-related; indeed, it is documented that immune-related genes have a tendency to display high variability, both for DNA sequence and expression levels, according to a red queen scenario. This point should be mentioned in the discussion.
     
   16. Line 378: It is not clear to me what the "overall success validation rate" refers to.
     
   17. Additional file 6B: Some instances of "bacground" should be replaced by "background".
     
   18. Additional file 6C: I do not understand how this table was built and used. Maybe a few more words would be helpful.
     
   19. Line 661: I could not find how the input was included in the analysis.
     
   20. M&M, Transcription start site detection: A regular RT-PCR was used, which does not seem suitable to identify TSS, contrary to a RACE-PCR approach for instance.
     
   21. Considering the reporter gene assays, when referring to "expression levels", the authors should clarify that they deal with RNA levels and not protein levels. Indeed, they are not always perfectly correlated and the presented experiments may be used for both measurements.

   22. In this study, the authors test the effects of TE insertions on Drosophila immune response using "only" one pathogen. As an opening, they may suggest that other results may be observed using other pathogens, since genetic interactions and pleiotropy are frequent regarding immune response, as suggested by the studies referenced in Table 1. Similarly, performing the same experiments using males instead of females may also lead to other results since genotype by sex interaction are observed in some pathogen response.
     
   23. Do the authors have clues about what makes a TE behave as a silencer or as an enhancer on the contrary? Is it possible to make predictions based on TE sequence, family or orientation relative to the gene ?
     
   24. When the number of measures is low, I suggest to show the actual values on the figures (in addition to or instead of boxplots). Boxplots are summaries that may be misleading in case there are too few data points.
     
   25. I suggest the use of RT-qPCR instead of qRT-PCR.
     
   26. Line 553: why do sex and tissue vary across experiments?
     
   27. Line 580: "°" should be removed.
     
   28. Optional suggestion: Would reaction norms be a relevant way to show the results of Figure 2?

**Reviewer 2**

Summary of the study
In this study, the authors identify polymorphic transposable elements insertions that may play a role in immune response in Drosophila melanogaster. They identify TEs found at high frequency (>10%) in several natural populations. Among these elements, 19 were found near 21 immune genes. The authors confirm the role of these genes in resistance to the pathogen Pseudomonas entomophila with several methods. Then, they measure allele-specific expression for 16 genes by identifying SNP in the coding sequence that are associated with the TE, and use this diagnostic marker to show that, during infection, alleles associated with the TE insertion are either overexpressed (3 genes) or underexpressed (5 genes) in two genetic backgrounds. TE silencing might lead to epigenetic modifications on the TE and its flanking sequences, but lower expression of alleles carrying the TE was not associated with epigenetic marks such as H3K9me3. On the other hand, cloning the promoter region with one of the candidate TEs (FBti0020057) in front of a reporter gene was associated with lower expression. The same design was used for four TEs associated with >1.5 fold higher expression of alleles associated with TEs insertions. Two of these candidates had an effect on the reporter gene expression. The authors then investigate in more details one of these two candidates, FBti0019386.
General comments
It is a very interesting study, and I am impressed by the amount of functional validation that was done. My expertise lies mostly in population genetics, a field that often identifies candidates for selection, but rarely validates them. This study makes a much-needed contribution to fill this gap in knowledge.

I have three main comments to make on this work, that I hope the authors will find constructive. Firstly, it is not easy for the reader to navigate between all these TEs, some of them being candidates for positive selection, some others being selected for further analyses because they show changes in expression. It would be nice to have a summary of all these TEs on a Figure rather than a Table like Table 1 in my opinion. Ultimately, after all the filtering, there are not that many TEs for which all lines of evidence converge to support a selective advantage (you run cloning experiments for FBti0020057, FBti0019386 and tdn8 if I understand correctly). I do agree that TEs are probably important, but we cannot yet say much about their relative importance compared to other types of variation.

Secondly, I was at first a bit puzzled by the very short length of the two insertions that you deem "representative" of under- and over-expression, FBti0020057 and FBti0019386. I like how you investigated the impact of FBti0019386. I am bit more sceptical for FBti0020057.
FBti0020057 is only 126bp long. You may want to discuss a bit more what in these 126bp could actually have a functional effect. I think that many readers expect that long TEs are more likely to have kept promotor/regulatory domains, while short TEs are often expected to have weaker impact on fitness. Does the TE contain a motif known to attract transcription factors for example? Or does it fall in an enhancer/promoter region?
At last, I had questions about the selection of candidate TEs for selection. I found that information about linkage between the TEs and neighbouring variants in natural populations could be made more substantial. You state that selected TEs lie in regions of high recombination, but it would be good to show to what extent allele frequencies of TEs and their neighbouring SNPs are correlated (for example through a LD decay analysis). I make more comments on this point below (see comment about L119).

Detailed comments:
L109: It might be useful to precise that you are using T-lex2, so you have some ascertainment bias here. If I understand correctly how the method works, it looks at presence/absence of TEs that are annotated in the reference genome. I understand that T-lex v1 had a de novo detection method, which is not in the second version.

L113: On the other hand, you will have more polymorphic near-neutral TEs in regions of high recombination, so more chances to get false positives. They will take more time to reach fixation because of the reduced effects of background selection/linked selection, but the allele frequency spectrum of polymorphic sites is less biased towards singletons in regions of high recombination. See also next comment.
L119: The main concern that I have here is that this 10% threshold seems a bit arbitrary and limited in power. Rech et al. used whole-genome resequencing data if I am correct. Ideally it would be nice to have a simple demographic model built from these data, and look at the distribution of frequencies for neutral markers. An easier way could be to use BAYPASS to estimate the population covariance matrix from SNPs, and use the script simulate.baypass() to generate neutral expectations. Or directly look at SNPs allele frequency spectra.
I understand that the strength of the paper lies in the functional analyses though, so the quickest way to proceed might be to look at haplotype extension around candidate TEs and compute the iHS score, for example using packages such as rehh. You also mention the nSL score in Table 1 (which is less sensitive to variations in recombination rates). If you have the VCF files it should be relatively straightforward. The advantage of this method is that you could give an estimate of how likely the TE is actually causal and not just linked to another causal locus. If the TE lies in the middle of an iHS peak, or really close to one, it would make the case for selection stronger. Alternatively, it might be that the haplotype not carrying the insertion is actually advantageous.


L123: What would be the random expectation though? Table 1 does not give information about the coordinates on Drosophila chromosomes so it is difficult to check at a glance whether these genes are all over the genomes or correspond to functional clusters

L159 (for the whole section): I wonder If you could not already present here elements that you discuss in the paragraph starting L382 (particularly sentence L389). Does the TE-associated allele induce an expression change in a direction consistent with higher fitness?
L207: I wonder whether a figure summarizing the findings in a manner similar to genome browsers could be useful. It could show the result of the iHS test for selection, along with histone marks, levels of gene and allele expression in different conditions, etc. There are nice R packages in BioConductor for this.
L216: Why is FBti0020057 representative? See also main comments.
L233: Maybe provide the effect size here, in addition to the p-value.
L414: It would be good to briefly explain how these data were generated.
L489. LINE-1, not INE-1.

**Authors’ response to reviewers**

Reviewer reports:

Reviewer #1: The article entitled "Regulatory regions in natural transposable element insertions drive interindividual differences in response to immune challenges in Drosophila" by Ullastres et al. reports the identification of polymorphic transposable element (TE) insertions that induce changes in immune gene transcript levels and allow an increase in the tolerance of Drosophila melanogaster to infection by Pseudomonas bacteria. This study is very interesting in the context that the determinism of natural variability in pathogen response is largely not understood.

Demonstrating a physiological role to a TE is usually not an easy task because of the difficulty to disentangle the role of the genetic background. In this article, using a variety of sophisticated molecular approaches (in vivo enhancer assays and CRISPR/Cas9 mediated deletions), the authors provide a clear demonstration that some TE insertions may participate in the variability observed regarding host response to pathogens. In addition, their results are very interesting because they illustrate the wideness of the spectrum of TE impacts on host genomes: from none to silencers or enhancers.

The aim and rationale are very clear, the methods are usefully very detailed, and all data are provided as Additional files. The methods are appropriate and all necessary controls are included. The conclusions are adequately supported by the data shown. This study is of interest for a broad audience in the genomics field.

I have a few questions and comments listed below.

1. I understand that TE insertions in regions with recombination rate = 0 were discarded (line 493); however, how were "regions with high recombination rate" (line 110) defined ? What threshold was used ?

>>>We first would like to thank the reviewer for their comments on our manuscript.

The reviewer is correct that we discarded regions with estimates of recombination rate = 0. This decision is based on the work of Haddrill et al 2007 (doi:10.1186/gb-2007-8-2-r18) that shows that reduced efficacy of selection is restricted to regions that lack crossing over. We thank the reviewer for pointing out to us that referring to the analyzed regions as “high recombination” is not correct. We now refer to the regions analyzed in our work as “regions with recombination rate larger than zero” and we have added the above mentioned reference to justify our choice.

2. Additional file 1B: I don't understand the discrepancy between "number of strains with the TE" and "number of PCR positive strains".

>>> We thank the reviewer for pointing this out to us. In the Additional File 1B, “number of strains with the TE” refers to the number of DGRP strains containing each insertion according to Rahman et al. (2015). On the other hand, “number of PCR positive strains” is the number of strains in which we have validated by PCR the presence of these insertions. We have now corrected the Additional File 1B to clarify this.

3. Additional file 1D, legend: "Functional (instead of funcional) information of nearby (instead of neaby) genes"

>>>Thanks for pointing this out. We have now corrected these typos.

4. Table 1: it would be nice also to tell whether TE insertions are full-length, and when they aren't, to which part of the sequence they correspond.

>>> We agree with the reviewer. We now indicate in Table 1 whether elements are full-length (FL) or truncated (T). When TEs are truncated we also indicate whether it corresponds to a solo LTR, a TIR region, or if the insertion is 5’ truncated.

5. Fig1 (and corresponding text): I find it a little misleading that upregulated and downregulated mutants are treated together. I suggest using separate panels or clearly labeling them.

>>>We have now added labels to each gene in Figure 1 so that it is clear which mutants are up-regulating, and which ones are down-regulating.

6. Additional file 3: I could not find the correspondence for the different backgrounds. I guess the solution is in Additional file 6A, but it should be clarified.

>>>We agree with the reviewer that the information on the backgrounds used should be included in this table. We have now added to Additional File 3 two columns indicating the fly strains used in every ASE experiment.

7. Figure 3B: labels are missing for the x axis.

>>>Thank you for pointing this out. We have now added the labels for the x axis in Figure 3B.

8. Line 202 "Candidate adaptive TEs associated… histone mark H3K9me3" section. The outputs of statistical tests are missing for the ChIP experiments.

From Figure 2, FBti0020057 is associated both with increased gene expression (CG15829) and decreased gene expression (CG8628); this should be clarified in this section. In addition, from fig 2, it appears that it is FBti0018868 that is associated with TM4SF; therefore, I don't understand why it is FBti0020057 that is tested with the promoter region of TM4SF in the reporter gene assay.

>>> Thank you for pointing that the statistical tests were missing. We have now added the p-values of all the analysis in the Figure 3 legend.

We have also clarified in Figure 2 that FBti0020057 is associated with lower allele-specific expression of CG8628, while it is also associated with increased allele-specific expression of CG15829.

We thank the reviewer for pointing out that FBti0020057 is not located nearby TM4SF. This was a typo. The in vivo enhancer assay for FBti0020057 was performed with the intergenic region between CG15829 and CG8628 genes with and without the insertion cloned in front of the reporter lacZ. We have now corrected this typo in our manuscript.

9. Line 221: why "two of the four"? From Fig2, five and three TEs are associated with high allele-specific expression in control and infection conditions, respectively.

>>> In this sentence, we refer to the four TEs tested in the in vivo enhancer assays. In these experiments, only two out of four tested TEs were acting as enhancers. We have now clarified in the title of this section and in the text that we refer to the TEs analyzed by enhancer assays.

10. Line 228: "promoter regions" : were these regions functionally validated as promoters or are they merely upstream, close to the genes? It is not an issue, but it should be clarified.

>>>We agree with the reviewer. By “promoter regions” we refer to regions located less than 1kb from a gene. We have now specified it in the text.

11. Figure 4B: half of the graph seems to be missing.

>>> Thanks for pointing this out to us. The graph is complete, however the schematic representation of the transgenic construct was incorrect. We have now modified the figure.

12. Figure 5C: Based on the text line 282, "flies with FBti0019386 overexpressed Bin1 compared to flies within this insertion (p-value < 0.001)". However, significance stars on the figure seem to correspond to the comparison Bin1 total vs red transcript.

>>>We apologize for this mistake. The reviewer is correct, significance stars correspond to the comparison of Bin1 total expression in flies with and without the TE. We have now corrected this.

13. ASE experiments: although the use of two different genetic backgrounds strengthens their conclusions, the authors should clearly mention that the insertion of the TE is not the only difference between the two alleles of the heterozygote, and therefore they cannot completely exclude that the TE insertion is not causative for the differences in expression levels. They explain that they have taken this issue into account in the M&M section (line 611), but this point deserves to be discussed more explicitly in the main article.

>>> We agree with the reviewer. We have now added this information to the main text also.

14. ASE experiments: I acknowledge that the number of genes is too small for statistical analyses, however, are there trends for associations of the direction of the change in gene expression with the position of the TE relative to the gene (eg upstream vs downstream, near vs far away, etc.) ? Or with the class of the TE ?

>>> We agree with the reviewer that with such small numbers we cannot do statistical analyses. As suggested by the reviewer, we have checked the class and the position regarding the nearby gene of TEs associated with gene up- and down-regulation. We found 3 LTRs, 2 nonLTRs and 2 DNA TEs associated with up-regulation and 2 LTRs, 3 nonLTR and 1 DNA TE associated with down-regulation. Regarding the position of the TE relative to the nearby gene, we found 3 TEs upstream, 2 TEs downstream, 2 TEs located in introns to be associated with up-regulation and 1 TE upstream, 2 TEs downstream, 3 TEs located in introns to be associated with down-regulation.

We did find that three TEs associated with up-regulation in infected conditions (tdn8, FBti0019386, and FBti0018868) were all LTR elements, and all of them were inserted upstream of the gene (FBti0019386 in the 5’UTR). Two of these TEs (tdn8 and FBti0019386) were proved to act as enhancers, and the presence of functional immune-related transcription factor binding sites was further demonstrated for FBti0019386. These results suggest that the sequence of the insertion and the position of the TE relative to the gene may have an impact in the nearby gene expression in at least infected conditions. Still, given the small numbers we prefer not talk about trends in our manuscript.

15. Line 336: "While these studies found… we found a significant higher proportion". Such a result may also be due to these genes being immune-related; indeed, it is documented that immune-related genes have a tendency to display high variability, both for DNA sequence and expression levels, according to a red queen scenario. This point should be mentioned in the discussion.

>>>We agree with the reviewer and we now mentioned in the discussion that there is evidence for fast evolution of immune-related genes. However, note that in Drosophila, a global survey of immune-related genes found that rapid adaptation of immune genes is the exception rather than the rule (Early et al 2017 10.1534/genetics.116.195016).

16. Line 378: It is not clear to me what the "overall success validation rate" refers to.

>>>We have now edited this section to clarify that we refer to the overall success validation rate of the TEs tested in the in vivo enhancer assays.

17. Additional file 6B: Some instances of "bacground" should be replaced by "background".

>>>Thank you for pointing this out. We have now removed this typo in Additional File 6B.

18. Additional file 6C: I do not understand how this table was built and used. Maybe a few more words would be helpful.

>>> We agree with the reviewer that the legend for additional file 6C was not clear. We have now added more information: “Analysis of the 1 kb upstream and downstream TE flanking regions to identify the sequences conserved between D. melanogaster and D. yakuba. The coordinates, length, % of conservation and location of the sequences conserved between D. melanogaster and D. yakuba according to VISTA are provided. ”

19. Line 661: I could not find how the input was included in the analysis.

>>> Thank you for pointing this out. We have added in the “Methods” section that the input was used as a reference value to normalize the immunoprecipitation enrichment in the RT-qPCR.

20. M&M, Transcription start site detection: A regular RT-PCR was used, which does not seem suitable to identify TSS, contrary to a RACE-PCR approach for instance.

>>> We agree with the reviewer that RT-PCR is not suitable for TSS identification. Because the presence of a TSS in FBti0019386 insertion was already reported by Batut et al (2013), we used RT-PCR to confirm that we could also detect a transcript containing the FBti0019386 insertion. We have now modified the Methods section to clarify this.

21. Considering the reporter gene assays, when referring to "expression levels", the authors should clarify that they deal with RNA levels and not protein levels. Indeed, they are not always perfectly correlated and the presented experiments may be used for both measurements.

>>> We agree with the reviewer and we have now clarified in the text that we refer to gene expression levels and not to protein levels.

22. In this study, the authors test the effects of TE insertions on Drosophila immune response using "only" one pathogen. As an opening, they may suggest that other results may be observed using other pathogens, since genetic interactions and pleiotropy are frequent regarding immune response, as suggested by the studies referenced in Table 1. Similarly, performing the same experiments using males instead of females may also lead to other results since genotype by sex interaction are observed in some pathogen response.

>>> We agree with the reviewer that these topics should be considered in the main text. We have mentioned them in the discussion section.

23. Do the authors have clues about what makes a TE behave as a silencer or as an enhancer on the contrary? Is it possible to make predictions based on TE sequence, family or orientation relative to the gene ?

>>> In the case of TEs acting as enhancers in response to infection conditions, we hypothesize that these could be related to the presence of binding sites for immune-related transcription factors. Indeed, we found that this seems to be the case for FBti0019386 insertion. We have now added the information on which TEs contain immune-related TFBS as reported in Villanueva-Cañas et al (2019) in Additional File 1E.

For TEs that behave as silencers, our first hypothesis was that they could be adding repressive histone marks. However, we were not able to validate this hypothesis for any of the six TEs tested. Alternatively, these TEs could be disrupting regulatory regions or could be adding regulatory regions that recruit transcriptional repressors. We have added this information to the manuscript.

As mentioned above, we think that the number of TEs analyzed is too small to arrive at any conclusion.

24. When the number of measures is low, I suggest to show the actual values on the figures (in addition to or instead of boxplots). Boxplots are summaries that may be misleading in case there are too few data points.

>>> We agree with the reviewer. We have now added the actual values as dots in all the boxplot figures.

25. I suggest the use of RT-qPCR instead of qRT-PCR.

We have now changed qRT-PCR for RT-qPCR.

26. Line 553: why do sex and tissue vary across experiments?

>>> For each mutant line, we performed the experiments in the tissue and sex where FlyBase database reported a higher expression of that particular gene. In the case where a gene is uniformly expressed across tissues, we extracted RNA from whole-flies. The detailed information about the sex and tissue analyzed for every mutant or RNAi line can be found in Additional File 2A. We agree with the reviewer that this needs to be explained. We have now added a sentence explaining why sex and tissue vary across experiments.

27. Line 580: "°" should be removed.

>>>Thank you for pointing this out. We have now removed this typo.

28. Optional suggestion: Would reaction norms be a relevant way to show the results of Figure 2 ?

>>> We would prefer to represent the results of the ASE experiments as they are currently depicted because the focus of our analysis is the comparison between flies with and without the insertions.

Reviewer #2: Summary of the study

In this study, the authors identify polymorphic transposable elements insertions that may play a role in immune response in Drosophila melanogaster. They identify TEs found at high frequency (>10%) in several natural populations. Among these elements, 19 were found near 21 immune genes. The authors confirm the role of these genes in resistance to the pathogen Pseudomonas entomophila with several methods. Then, they measure allele-specific expression for 16 genes by identifying SNP in the coding sequence that are associated with the TE, and use this diagnostic marker to show that, during infection, alleles associated with the TE insertion are either overexpressed (3 genes) or underexpressed (5 genes) in two genetic backgrounds. TE silencing might lead to epigenetic modifications on the TE and its flanking sequences, but lower expression of alleles carrying the TE was not associated with epigenetic marks such as H3K9me3. On the other hand, cloning the promoter region with one of the candidate TEs (FBti0020057) in front of a reporter gene was associated with lower expression. The same design was used for four TEs associated with >1.5 fold higher expression of alleles associated with TEs insertions. Two of these candidates had an effect on the reporter gene expression. The authors then investigate in more details one of these two candidates, FBti0019386.

General comments

It is a very interesting study, and I am impressed by the amount of functional validation that was done. My expertise lies mostly in population genetics, a field that often identifies candidates for selection, but rarely validates them. This study makes a much-needed contribution to fill this gap in knowledge.

I have three main comments to make on this work, that I hope the authors will find constructive. Firstly, it is not easy for the reader to navigate between all these TEs, some of them being candidates for positive selection, some others being selected for further analyses because they show changes in expression. It would be nice to have a summary of all these TEs on a Figure rather than a Table like Table 1 in my opinion.

>>> We agree with the reviewer and we have now added a new Figure (Figure 6, see below) that summarizes the results obtained in the different experiments (ASE, in vivo enhancer assays, H3k9me3 enrichment, etc.) for all the 14 TEs that were found to be associated with allele specific expression changes.

Ultimately, after all the filtering, there are not that many TEs for which all lines of evidence converge to support a selective advantage (you run cloning experiments for FBti0020057, FBti0019386 and tdn8 if I understand correctly). I do agree that TEs are probably important, but we cannot yet say much about their relative importance compared to other types of variation.

> We agree with the reviewer and that’s why we do not make comparative claims on the relative importance of TEs. We do think (and show) that they do play a role and as such should be taken into account when analyzing the genetic basis of any trait.

Secondly, I was at first a bit puzzled by the very short length of the two insertions that you deem "representative" of under- and over-expression, FBti0020057 and FBti0019386. I like how you investigated the impact of FBti0019386. I am bit more sceptical for FBti0020057.

>>> This is a misunderstanding. We did not investigate the impact of FBti0020057 because we considered it “representative”. We performed the in vivo enhancer assays with this insertion as an example to show that although we did not find evidence of histone marks, these TEs are likely causal mutations for the observed down-regulation and the molecular mechanism is yet to be identified. We have now removed the word “representative” and clarified that it is an example in the text.

FBti0020057 is only 126bp long. You may want to discuss a bit more what in these 126bp could actually have a functional effect. I think that many readers expect that long TEs are more likely to have kept promotor/regulatory domains, while short TEs are often expected to have weaker impact on fitness. Does the TE contain a motif known to attract transcription factors for example? Or does it fall in an enhancer/promoter region?

>>> In the particular case of FBti0020057, this insertion is indeed located in the promoter region of CG8628 and we show that the promoter region with the TE reduces the expression of the reporter gene in the reporter assay. We have now added the hypothesis that the TE might be disrupting the promoter to the main text. We have also added an Additional File (Additional File 1E) with the analysis of transcription factor binding sites for each TE tested in the ASE experiment.

Moreover, despite being only 126bp, FBti0020057 harbors one immune-related binding site for the Nub transcription factor, which negatively regulates many genes that are involved in immune and stress responses, while it is a positive regulator of genes involved in differentiation and metabolism (Dantoft 2013).

At last, I had questions about the selection of candidate TEs for selection. I found that information about linkage between the TEs and neighbouring variants in natural populations could be made more substantial. You state that selected TEs lie in regions of high recombination, but it would be good to show to what extent allele frequencies of TEs and their neighbouring SNPs are correlated (for example through a LD decay analysis). I make more comments on this point below (see comment about L119).

>>>We would like to highlight that, as mentioned in the manuscript, the analysis for evidence of selection in the regions flanking the TE insertions are not results obtained in this work. We are reporting results from the literature, some of them from our lab but also from other authors, because we think it is relevant to point out which of the analyzed insertions have previous evidence for their potential role in adaptive evolution.

From our initial list of 19 candidate TEs, 16 of them have been investigated for evidence of selection. We have now added the information on which TEs have been previously investigated to Table 1. Seven of these TEs have evidence of selection, as was already reported in Table 1. However, only for one of them, FBti0019386, the authors explicitly mentioned that “No other polymorphisms were found that could potentially confound the results of our tests of selection suggesting that the TE is the causative mutation” (Ullastres et al 2015).

We have further investigated the patterns reported in Rech et al (2019) for three TEs included in our analyses: FBti0019457 and FBti0020057 that showed evidence of selection in North American populations and FBti0061506 that showed evidence of selection in European populations. We did not find any other SNP in perfect linkage with the TE in the strains analyzed. However, we agree with the reviewer that we cannot completely discard that other mutations beside the TE are affecting gene expression. We now mention this possibility more explicitly in our manuscript.

Detailed comments:

L109: It might be useful to precise that you are using T-lex2, so you have some ascertainment bias here. If I understand correctly how the method works, it looks at presence/absence of TEs that are annotated in the reference genome. I understand that T-lex v1 had a de novo detection method, which is not in the second version.

>>>We agree with the reviewer that we should state that our screening is mostly based in reference TE insertions. We have added this information to the results section of the manuscript.

We are involved in the development of T-lex, and none of the versions included a de novo detection method. We intended to include a module for de novo detection and a preliminary version of it might even have been presented at some conference, but it was never finally included in any of the three versions available for T-lex.

L113: On the other hand, you will have more polymorphic near-neutral TEs in regions of high recombination, so more chances to get false positives. They will take more time to reach fixation because of the reduced effects of background selection/linked selection, but the allele frequency spectrum of polymorphic sites is less biased towards singletons in regions of high recombination. See also next comment.

L119: The main concern that I have here is that this 10% threshold seems a bit arbitrary and limited in power. Rech et al. used whole-genome resequencing data if I am correct. Ideally it would be nice to have a simple demographic model built from these data, and look at the distribution of frequencies for neutral markers. An easier way could be to use BAYPASS to estimate the population covariance matrix from SNPs, and use the script simulate.baypass() to generate neutral expectations. Or directly look at SNPs allele frequency spectra.

I understand that the strength of the paper lies in the functional analyses though, so the quickest way to proceed might be to look at haplotype extension around candidate TEs and compute the iHS score, for example using packages such as rehh. You also mention the nSL score in Table 1 (which is less sensitive to variations in recombination rates). If you have the VCF files it should be relatively straightforward. The advantage of this method is that you could give an estimate of how likely the TE is actually causal and not just linked to another causal locus. If the TE lies in the middle of an iHS peak, or really close to one, it would make the case for selection stronger. Alternatively, it might be that the haplotype not carrying the insertion is actually advantageous.

>>>The reviewer is correct that the threshold is arbitrary. However, we do mention in the material and methods section that varying this threshold does not substantially alter the number of TEs considered to be at high frequencies and thus likely to be adaptive. This is also consistent with results obtained in previous works (see González et al 2010: doi:10.1371/journal.pgen.1000905). As the reviewer recognizes, we believe that the strength of our work is on the experimental validation of the TE candidates and we indeed showed that a substantial proportion of our candidates are associated with changes in the expression of nearby genes. Furthermore, TEs that have been functionally validated cover the spectrum of frequencies that we consider as potentially adaptive: from a population frequency of ~10% in the case of FBti0019985 (this work and also Merenciano et al 2016) to more intermediate frequencies, e.g. tdn8 is present at 31% frequency and FBti0019386 is present at 45% to 65% population frequencies, to very high population frequencies such as FBti0020057 that is present at population frequencies ranging from 64% to 93%.

We did not perform scans for selection in our manuscript, but rather report information already available in the literature. Still, we have followed the reviewer suggestion and as mentioned above, for four of the seven TEs for which evidence of selection was provided in the literature, either the authors already mentioned that the TE was the most likely causal mutations or we were not able to find any other polymorphism in complete linkage disequilibrium with the TE insertion, suggesting that the TE is the causal mutation. We agree with the reviewer that we cannot completely discard that other polymorphisms can also be affecting the patterns of selection. We have made this more clear in the revised version of our manuscript.

L123: What would be the random expectation though? Table 1 does not give information about the coordinates on Drosophila chromosomes so it is difficult to check at a glance whether these genes are all over the genomes or correspond to functional clusters.

>>> We agree with the reviewer, and we have now added to Table 1 a column with the chromosome and the start coordinate for all the TEs to clarify that they are present in all chromosomes and not in functional clusters.

L159 (for the whole section): I wonder If you could not already present here elements that you discuss in the paragraph starting L382 (particularly sentence L389). Does the TE-associated allele induce an expression change in a direction consistent with higher fitness?

>>> We agree with the reviewer and we now also mention in the results section that the direction of the change of expression is consistent with higher fitness.

L207: I wonder whether a figure summarizing the findings in a manner similar to genome browsers could be useful. It could show the result of the iHS test for selection, along with histone marks, levels of gene and allele expression in different conditions, etc. There are nice R packages in BioConductor for this.

>>> We agree with the reviewer that a figure summarizing the results for all TEs is needed. We have added a figure (Figure 6) in which we represent all the analysis performed for each TE tested in the ASE experiments and the results obtained. We have also highlighted the statistically significant results.

L216: Why is FBti0020057 representative? See also main comments.

>>> In this case, we reported the in vivo enhancer assay results for FBti0020057 as an example reporting that the TE sequence reduces the expression of the reporter gene probably due to the recruitment of repressors or by disrupting the gene promoter region rather than by recruiting repressive histone marks. We have now removed the word “representative” to clarify this.

L233: Maybe provide the effect size here, in addition to the p-value.

>>> We agree with the reviewer and we have added the effect size to the main text.

L414: It would be good to briefly explain how these data were generated.

>>> The estimation of TE frequencies annotated in the reference genome using the DGRP strains is explained in the paragraph “Transposable elements dataset” within the Methods section. We have now redirected the reader to that section in the text.

L489. LINE-1, not INE-1.

>>> We do refer to INE-1 and not LINE-1. INE-1 is a family of highly abundant DNA TEs present in Drosophila.

**Second round of review**

**Reviewer 1**

I thank the authors for this revised version of the article, which answers all my comments.

**Reviewer 2**

I am happy with the explanations and changes made by the authors. I still believe that a more systematic and comprehensive assessment of selection signals is needed in this model system, but this issue is tangential to the work presented here. I really appreciate the detailed functional analysis, which must have been a lot of work. It will certainly inform future evolutionary studies aiming at understanding the fitness effects of TEs in Drosophila and beyond.
